# Aneurysm at an Extremely Proximal Posterior Communicating Artery Origin Treated With Flow Diverter-Assisted Coiling: A Case Report

**DOI:** 10.7759/cureus.110919

**Published:** 2026-06-15

**Authors:** Junichi Takeda, Ichi Ly, Takehiro Suyama, Ryosuke Matsuda, Masahiro Nonaka

**Affiliations:** 1 Neurosurgery, Kansai Medical University, Hirakata, JPN

**Keywords:** flow-diverter stent, internal carotid artery (ica), paraclinoid aneurysm, pipeline flex embolization device, posterior communicating artery (pcom)

## Abstract

The posterior communicating artery (Pcom) arises from the supraclinoid internal carotid artery (ICA), just proximal to the anterior choroidal artery. Although caliber variations of the Pcom are common, marked proximal displacement of its origin is extremely rare. We report a case of an aneurysm initially diagnosed as a paraclinoid ICA aneurysm that was subsequently identified as a branch-incorporated internal carotid-posterior communicating artery (IC-PC) aneurysm associated with an aberrant Pcom arising from the ICA siphon proximal to the ophthalmic artery.

A man in his 60s underwent magnetic resonance imaging/magnetic resonance angiography for headache evaluation, which revealed a 7.5-mm saccular aneurysm in the left paraclinoid ICA. Digital subtraction angiography and three-dimensional rotational angiography demonstrated that a branch arising from the aneurysm base coursed posteriorly and continued to the P1/P2 segment of the posterior cerebral artery. An Allcock test confirmed retrograde opacification of this vessel and the aneurysm from the posterior circulation. These findings established the diagnosis of an aneurysm incorporating an extremely proximal Pcom rather than a simple paraclinoid ICA aneurysm. Because another aneurysm with an irregular shape was also present at the ipsilateral ICA-anterior choroidal artery junction, flow diversion was selected to treat both lesions simultaneously. After dual antiplatelet therapy, Pipeline Flex placement with adjunctive coil embolization was performed successfully.

The postoperative course was uneventful. Follow-up magnetic resonance angiography at six months showed complete disappearance of the intra-aneurysmal signal. This case highlights the importance of meticulous preoperative vascular assessment in distinguishing rare branch-incorporated aneurysms from paraclinoid ICA aneurysms and in planning safe endovascular treatment.

## Introduction

The posterior communicating artery (Pcom) typically arises from the supraclinoid segment of the internal carotid artery (ICA), usually just proximal to the origin of the anterior choroidal artery. Variations in Pcom caliber, including hypoplasia and fetal-type posterior cerebral artery, are well recognized. In contrast, marked proximal displacement of the Pcom origin is exceptionally rare [1-3]. Uchino et al. recently reported an “extreme proximal” Pcom arising from the paraclinoid ICA just distal to the ophthalmic artery [1].

Several anomalous vascular configurations involving the supraclinoid ICA and Pcom have been described, including duplicated Pcom and supraclinoid ICA fenestration associated with aneurysm formation [4-6]. These anomalies are considered to reflect developmental variations of the primitive carotid-basilar anastomotic system and may predispose to aneurysm development because of altered hemodynamic stress and vascular wall fragility [4,5]. However, aneurysms arising from an anomalously proximal Pcom origin remain extremely uncommon, and their optimal treatment strategy has not been established because of the limited number of reported cases.

We report a case that was initially interpreted as a left paraclinoid ICA aneurysm but was ultimately diagnosed as an internal carotid-posterior communicating artery (IC-PC) aneurysm incorporating an anomalously low-origin Pcom arising from the ICA siphon proximal to the ophthalmic artery. The lesion was successfully treated with flow diverter-assisted coil embolization, and follow-up angiography confirmed complete occlusion without ischemic complications. This case highlights the importance of detailed angiographic evaluation for accurate anatomical diagnosis and suggests that flow diverter-assisted treatment may be a feasible therapeutic option for this rare vascular entity.

## Case presentation

History and preoperative findings

A man in his 60s with a history of hypertension underwent brain magnetic resonance imaging/magnetic resonance angiography (MRI/MRA) for evaluation of headache. He had no neurological deficits and no family history of cerebral aneurysms.

MRA revealed a 7.5-mm saccular aneurysm at the left paraclinoid ICA, and the lesion was initially considered a paraclinoid ICA aneurysm (Figure 1).

**Figure 1:**
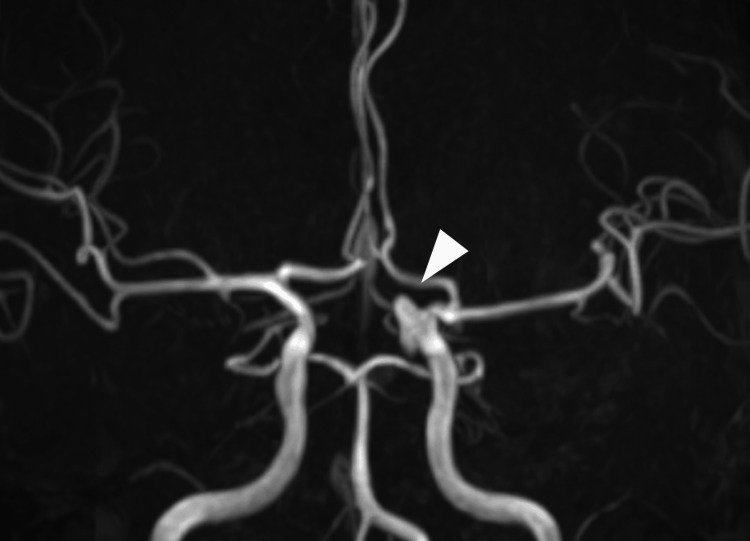
Preoperative magnetic resonance angiography (MRA) Preoperative MRA demonstrates a saccular aneurysm 7.5 mm in maximum diameter in the left C5 segment of the internal carotid artery (arrowhead). The lesion was initially considered a paraclinoid ICA aneurysm.

However, subsequent digital subtraction angiography demonstrated that the aneurysm projected medially from the ICA siphon, and a branch arising from the aneurysm base coursed posteriorly toward the posterior cerebral artery. Three-dimensional rotational angiography showed continuity of this branch with the P1/P2 segment. In addition, a left Allcock test demonstrated retrograde filling of the vessel and opacification of the aneurysm from the posterior circulation. These findings indicated that the branch was an anomalous Pcom arising from the ICA siphon at a point even more proximal than the origin of the ophthalmic artery (Figure 2). The lesion was therefore diagnosed as a branch-incorporated IC-PC aneurysm rather than a simple paraclinoid ICA aneurysm. An additional 3.5-mm irregularly shaped aneurysm was also identified at the ipsilateral ICA-anterior choroidal artery junction. Because both lesions could be treated within the same reconstructive strategy, flow diversion was selected. Because the aneurysm incorporated the origin of the aberrant Pcom, the likelihood of complete aneurysm occlusion with flow-diverter treatment alone was considered lower. Therefore, adjunctive coil embolization was performed in addition to flow-diverter placement.

**Figure 2:**
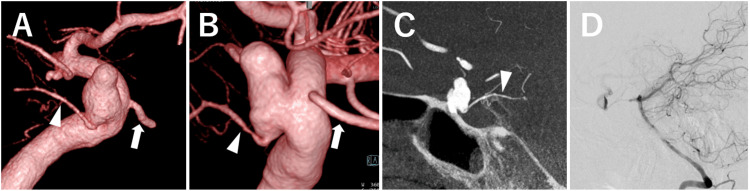
Preoperative three-dimensional rotational angiography and Allcock test. Preoperative three-dimensional rotational angiography (A, B) demonstrates a branch arising from the aneurysm base and coursing posteriorly (arrowhead), as well as the ophthalmic artery (arrow). A small aneurysm is also seen at the anterior choroidal artery origin. A sagittal slab maximum intensity projection image (C) shows that the branch courses over the posterior clinoid process and continues into the posterior circulation (arrowhead). On lateral vertebral angiography during the Allcock test (D), this branch was identified as the posterior communicating artery.

Endovascular treatment

Dual antiplatelet therapy with aspirin 100 mg and prasugrel 3.75 mg was initiated 10 days before treatment. The procedure was performed under general anesthesia via a transfemoral approach. After systemic heparinization with a target activated clotting time of >250 seconds, an 8-Fr Optimo® guiding catheter (Tokai Medical Products, Aichi, Japan) was positioned in the left ICA, and a 6-Fr Navien® intermediate catheter (Medtronic, Minneapolis, MN, USA) was advanced into the petrous ICA. A Phenom 27 microcatheter (Medtronic) was navigated to the distal M1 segment, and a Phenom 17 microcatheter (Medtronic) was placed within the aneurysm sac. A Pipeline Flex® 5 × 18 mm flow diverter (Medtronic) was deployed from the M1 segment to the cavernous segment of the ICA. Given the branch-incorporated configuration of the aneurysm, adjunctive coiling was performed with two Axium Prime coils (Medtronic). Cone-beam CT confirmed good wall apposition of the flow diverter. Final angiography demonstrated marked reduction of intra-aneurysmal flow with preserved visualization of the anomalous Pcom (Figure 3).

**Figure 3:**
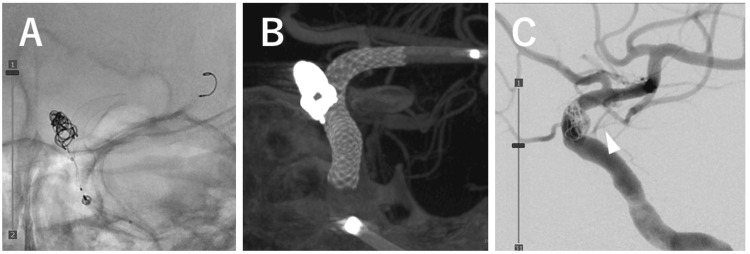
Intraoperative angiography. Intraoperative angiography (A, B) shows deployment of a Pipeline flex flow diverter from the middle cerebral artery to the C4 segment of the internal carotid artery, with adjunctive coil embolization performed within the aneurysm. Final angiography (C) demonstrates preservation of the anomalously originating posterior communicating artery (Pcom) (arrowhead).

Postoperative course

The postoperative course was uneventful. No new neurological deficits developed, and the patient was discharged home on postoperative day 5. Serial follow-up MRI after treatment demonstrated neither the development of neurological symptoms nor the appearance of ischemic lesions. MRA at six months after treatment showed complete disappearance of the intra-aneurysmal signal (Figure 4).

**Figure 4:**
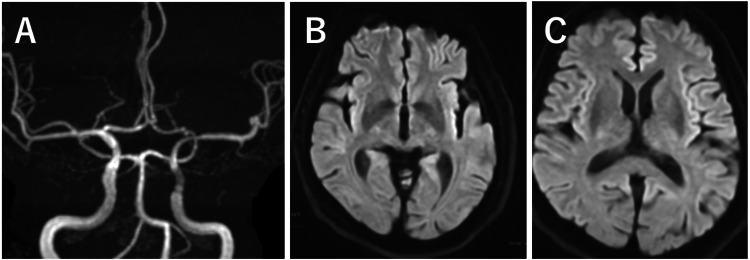
Follow-up magnetic resonance angiography (MRA) and diffusion-weighted imaging at six months. Follow-up MRA obtained six months after treatment (A) shows disappearance of the intra-aneurysmal signal. Diffusion-weighted imaging (B, C) demonstrates no apparent ischemic complications.

## Discussion

This variant also had direct diagnostic implications. The lesion was initially interpreted as a typical paraclinoid ICA aneurysm without an associated branch vessel, but detailed angiographic assessment demonstrated that it was actually a branch-incorporated aneurysm arising at the origin of an anomalous Pcom. Murai et al. reported a similar diagnostic pitfall in which an aneurysm thought to be paraclinoid was ultimately shown to originate from an anomalous Pcom origin [3]. Our case extends that concept to an even more proximal arterial origin.

A notable feature of the present case was the extremely rare anatomical variation in which the Pcom originated from the ICA siphon proximal to the origin of the ophthalmic artery. Uchino et al. reported in 2025 an “extreme proximal” Pcom arising from the paraclinoid ICA just distal to the origin of the ophthalmic artery and stated that no similar cases had previously been reported [1]. However, in the present case, the Pcom arose from an even more proximal site, namely proximal to the origin of the ophthalmic artery, and to the best of our knowledge, no previous report has described a Pcom originating from such a proximal location. Embryologically, this anomaly may be explained by regression of the distal portion of a small channel in a supraclinoid ICA fenestration, particularly Haryu’s type C variant, in which a hypoplastic fenestration joins the Pcom itself [1,2].

The relationship between fenestration-related arterial anomalies and aneurysm formation may also be relevant. Haryu et al. found a high frequency of aneurysm association in supraclinoid ICA fenestration, particularly in lesions involving the Pcom-side channel [2]. Baba et al. likewise described an aneurysm associated with fenestration of the Pcom itself [4]. Although causality cannot be proven in a single case, abnormal embryologic vessel fusion and altered local hemodynamics may have contributed to aneurysm development in our patient.

Flow diversion across the Pcom origin remains a matter of anatomical context. Prior studies have shown that coverage of the Pcom may lead to reduced flow or even occlusion, especially when the ipsilateral P1 segment is well developed, yet ischemic sequelae are uncommon in such settings [5-7]. In contrast, fetal-type Pcom aneurysms are known to have lower occlusion rates after flow diversion because persistent demand through the Pcom may prevent effective aneurysm thrombosis [8-10]. In our patient, the P1 segment was sufficiently preserved, and the anomalous Pcom ultimately regressed without ischemic complications, supporting the hemodynamic rationale for flow diversion in this particular anatomy. Previous computational fluid dynamics (CFD) studies of paraophthalmic and paraclinoid aneurysms have demonstrated marked reductions in intra-aneurysmal velocity and wall shear stress after flow-diverter placement, supporting the hemodynamic mechanism of aneurysm occlusion [11]. However, to the best of our knowledge, no CFD studies have specifically evaluated aneurysms associated with such a rare aberrant Pcom origin as observed in the present case.

## Conclusions

We reported a rare IC-PC aneurysm with an aberrant Pcom origin located at the paraophthalmic segment. This lesion can mimic a typical paraclinoid ICA aneurysm on noninvasive imaging. Careful angiographic evaluation, including assessment of branch continuity and collateral filling, is essential for correct diagnosis. In selected cases with a preserved P1 segment, flow diverter-assisted coil embolization may be an effective treatment strategy.
